# Supplementary material for: Resting Energy Expenditure Is Elevated in Asthma
Source: Nutrients. 2021 Mar 25;13(4):1065. doi: 10.3390/nu13041065 (PMC8064324; doi:10.3390/nu13041065)
Supplement: Supplementary file 1 [file nutrients-13-01065-s001.pdf]

Online Data Supplement

Resting Energy Expenditure is Elevated in Asthma

Jacob T. Mey[1,2], Brittany Matuska[2], Laura Peterson[2], Patrick Wyszynski[2], Michelle Koo[2], Jacqueline Sharp[2], Emily Pennington[3], Stephanie McCarroll[3], Sarah Micklewright[3], Peng Zhang[3], Mark Aronica[2,3], Kristin K. Hoddy[1], Catherine M. Champagne[1], Steven B. Heymsfield[1], Suzy A.A. Comhair[2], John P. Kirwan[1,2], Serpil C. Erzurum[2,3], Anny Mulya[2]

[1] Pennington Biomedical Research Center, Baton Rouge, LA

[2] Inflammation and Immunity, Lerner Research Institute, Cleveland Clinic, Cleveland OH

[3] Respiratory Institute, Cleveland Clinic, Cleveland, OH

TABLE S1. REE Prediction Equations

Mifflin-St. Jeor

Men:

REE = 10 x weight (kg) + 6.25 x height (cm) – 4.92 x age (y) + 5.

Women:

REE = 10 x weight (kg) + 6.25 x height (cm) – 4.92 x age (y) - 161.

Harris-Benedict

Men:

REE = 66.47+13.75 x weight (kg) + 5.0 x height (cm) - 6.75 x age (y).

Women:

REE = 665.09 + 9.56 x weight (kg) +1.84 x height (cm) -4.67 x age (y).

WHO

Men:

If 18-30 (y); REE = 15.4 x weight (kg) – 27 x height (m) + 717.
If 31-60 (y); REE = 11.3 x weight (kg) + 16 x height (m) + 901.
If >60 (y); REE = 8.8 x weight (kg) + 1,128 x height (m) - 1,071.

Women:

If 18-30 (y); REE = 13.3 x weight (kg) + 334 x height (m) + 35.
If 31-60 (y); REE = 8.7 x weight (kg) – 25 x height (m) + 865.
If >60 (y); REE = 9.2 x weight (kg) + 637 x height (m) – 302.

Oxford

(A conversion factor of 238.846 was added to convert MJ/day to kcal/day.)

Men:

If 18-30 (y); REE = 0.0669 x weight (kg) + 2.28
If 31-60 (y); REE = 0.0592 x weight (kg) + 2.48
If >60 (y); REE = 0.0563 x weight (kg) + 2.15

Women:

If 18-30 (y); REE = 0.0546 x weight (kg) + 2.33
If 31-60 (y); REE = 0.0407 x weight (kg) + 2.90
If >60 (y); REE = 0.0424 x weight (kg) + 2.38

Table S2. Clinical Characteristics and Comorbidities

	Control (n=20)	Asthma (n=41)	*P* value
Doctor diagnosis of asthma	0 (0)	41 (100)	<0.01
Pre- vs. post-puberty onset		19 (46) vs. 22 (54)	
Smoking history	7 (35)	10 (24)	0.54
Nasal polyp	0 (0)	11 (27)	0.01
Chronic or recurrent sinusitis	1 (2)	10 (24)	0.08
Pneumonia history	4 (20)	18 (44)	0.09
Bronchitis history	10 (50)	32 (78)	0.04
Emphysema or COPD	0 (0)	1 (2)	1.00
Snoring	9 (45)	14 (34)	0.57
Breathing sleep disorder	2 (10)	6 (15)	1.00
Allergy	7 (35)	33 (80)	0.04
Hypertension	0 (0)	3 (7)	0.54
Chronic Kidney Disease	0 (0)	1 (2)	1.00
Post Menopause	2 (10)	7 (17)	1.00

Data are n (%)

P values denoted the comparison between healthy control and asthmatic population as analyzed by Fisher Exact Test. COPD, chronic obstructive pulmonary disease.

Table S3. Asthma Control Test (ACT) Score of Patients with Asthma

Test Component	Mean ± SD
**Missing work due to asthma**	**4.4 ± 0.9**
All of the time – n (%)	1 (2)
Some of the time – n (%)	5 (12)
A little of the time – n (%)	9 (22)
None of the time – n (%)	26 (63)
**Shortness of breath**	**4.0 ± 1.0**
More than once per day – n (%)	2 (5)
Once per day – n (%)	1 (2)
3 – 6 times per week – n (%)	6 (15)
Once or twice a week – n (%)	20 (49)
Not at all – n (%)	12 (29)
**Sleep disturbances due to asthma**	**4.3 ± 1.0**
4 or more nights a week – n (%)	1 (2)
2 – 3 nights a week – n (%)	4 (10)
Once a week – n (%)	3 (7)
Once or twice a week – n (%)	11 (27)
Not at all – n (%)	22 (54)
**Necessity to use rescue medication**	**3.7 ± 1.4**
3 or more times per day – n (%)	4 (10)
1 – 2 times per day – n (%)	7 (17)
2 – 3 times per week – n (%)	4 (10)
Once a week or less – n (%)	8 (20)
Not at all – n (%)	18 (44)
**Self-assessment of asthma control**	**4.1 ± 0.9**
Poorly controlled – n (%)	2 (5)
Somewhat controlled – n (%)	9 (22)
Well controlled – n (%)	13 (32)
Completely controlled – n (%)	17 (41)
**Total ACT Score**	**20.4 ± 4.4**
Subject with ACT score ≤ 19 – n (%)	12 (29)

The Asthma Control Test Score was assessed over the past 4 weeks prior to exam.

Table S4. Asthma-related Medications

**Medications**	**Asthma (n=41)**
**Short Acting Beta Agonist – n (%)**	**29 (71%)**
**Short Acting Muscarinic Agonist – n (%)**	**1 (2%)**
**Inhaled steroids – n (%)**	**24 (58%)**
Fluticasone/propionate – n	2
Fluticasone/salmeterol – n	5
Mometasone – n	5
Budesonide/formoterol – n	5
Mometasone/formoterol – n	5
Budesonide – n	2
**Oral steroids – n (%)**	**2 (5%)**
**Biologicals – n (%)**	**4 (10%)**
Omalizumab – n	1
Mepolizumab – n	2
Benralizumab – n	1
**Long acting muscarinic antagonist – n (%)**	**2 (5%)**
Tiotropium – n	1
Aclidinium – n	1
**Leukotriene Modifier – n (%)**	**15 (37%)**
Montelukast – n	15

TABLE S5. Detail Summary Statistics for REE prediction model

Model 1 – using previously known criteria (age (years), sex (male=1; female=0), fat mass (kg) and fat free mass (kg))

Regression Statistics	
Multiple R	0.763
R square	0.583
Adjusted R square	0.552
Standard Error	225.891
Observations	60

ANOVA

	df	*SS*	*MS*	*F*	*Significance F*
Regression	4	3918522	979630	19.198	6.22E-10
Residual	55	2806473	51027		
Total	59	6724994			

	Coefficients	*Standard Error*	*t Stat*	*P value*	*Lower 95%*	*Upper 95%*
Intercept	350.5	234.7	1.493	0.141	-119.9	820.9
Age (years)	-0.1	2.9	-0.042	0.966	-5.9	5.6
Sex	27.9	116.3	0.240	0.811	-205.2	261.0
Fat mass (kg)	11.4	3.3	3.472	0.001	4.8	18.0
Fat-free mass (kg)	18.4	4.9	3.753	0.000	8.6	28.2

Model 2 – addition of asthma status (asthma diagnosis=1; no asthma diagnosis=0) to Model 1

Regression Statistics	
Multiple R	0.769
R square	0.592
Adjusted R square	0.554
Standard Error	225.538
Observations	60

ANOVA

	df	*SS*	*MS*	*F*	*Significance F*
Regression	5	3978152	795630	15.641	1.69E-09
Residual	54	2746842	50867		
Total	59	6724994			

	Coefficients	*Standard Error*	*t Stat*	*P value*	*Lower 95%*	*Upper 95%*
Intercept	373.1	235.3	1.586	0.119	-98.7	844.8
Age (years)	-0.4	2.9	-0.156	0.876	-6.2	5.3
Sex	62.7	120.5	0.520	0.605	-178.9	304.3
Fat mass (kg)	10.5	3.4	3.105	0.003	3.7	17.3
Fat-free mass (kg)	17.4	5.0	3.508	0.001	7.5	27.4
Asthma	73.9	68.2	1.083	0.284	-62.9	210.6

Model 3 – addition of FENO (ppm) value to Model 1

Regression Statistics	
Multiple R	0.775
R square	0.600
Adjusted R square	0.563
Standard Error	223.100
Observations	60

ANOVA

	df	*SS*	*MS*	*F*	*Significance F*
Regression	5	4037211	807442	16.222	9.59E-10
Residual	54	2687783	49774		
Total	59	6724994			

	Coefficients	*Standard Error*	*t Stat*	*P value*	*Lower 95%*	*Upper 95%*
Intercept	361.6	231.9	1.559	0.125	-103.4	826.6
Age (years)	-1.5	3.0	-0.510	0.612	-7.5	4.4
Sex	6.8	115.7	0.059	0.954	-225.2	238.7

Fat mass (kg)	11.6	3.3	3.569	0.001	5.1	18.1
Fat-free mass (kg)	18.4	4.8	3.798	0.000	8.7	28.1
FENO (ppm)	1.9	1.2	1.544	0.128	-0.6	4.3

Model 4 – addition of white blood cell count (WBC, millions of cells) value to Model 1

Regression Statistics	
Multiple R	0.789
R square	0.623
Adjusted R square	0.588
Standard Error	216.634
Observations	60

ANOVA

	df	*SS*	*MS*	*F*	*Significance F*
Regression	5	4190767	838153	17.860	2.06E-10
Residual	54	2534228	46930		
Total	59	6724994			

	Coefficients	*Standard Error*	*t Stat*	*P value*	*Lower 95%*	*Upper 95%*
Intercept	177.4	236.3	0.751	0.456	-296.3	651.2
Age (years)	-1.0	2.8	-0.346	0.731	-6.5	4.6
Sex	31.9	111.6	0.286	0.776	-191.7	255.6
Fat mass (kg)	8.4	3.4	2.489	0.016	1.6	15.2
Fat-free mass (kg)	19.2	4.7	4.068	0.000	9.7	28.6
WBC (x10^6 cells)	45.3	18.8	2.409	0.019	7.6	83.0

Model 5 – addition of neutrophil count (millions of cells) value to Model 1

Regression Statistics	
Multiple R	0.785
R square	0.616
Adjusted R square	0.580
Standard Error	218.726
Observations	60

ANOVA

	df	*SS*	*MS*	*F*	*Significance F*
Regression	5	4141578	828316	17.314	3.41E-10
Residual	54	2583416	47841		
Total	59	6724994			

	Coefficients	*Standard Error*	*t Stat*	*P value*	*Lower 95%*	*Upper 95%*
Intercept	259.7	231.1	1.1213	0.266	-203.8	723.1
Age (years)	-1.2	2.8	-0.419	0.677	-6.8	4.5
Sex	21.7	112.7	0.193	0.848	-204.1	247.6
Fat mass (kg)	9.0	3.4	2.653	0.010	2.2	15.7
Fat-free mass (kg)	19.2	4.8	4.031	0.000	9.6	28.7
Neutrophil (x10^6 cells)	48.3	22.4	2.159	0.035	3.5	93.2

Model 6 – addition of FVC (% predicted) to Model 1

Regression statistics	
Multiple R	0.767
R square	0.589
Adjusted R square	0.551
Standard Error	226.337
Observations	60

ANOVA

	df	*SS*	*MS*	*F*	*Significance F*
Regression	5	3958654	791731	15.455	2.03E-09
Residual	54	2766340	51229		
Total	59	6724994			

	Coefficients	*Standard Error*	*t Stat*	*P value*	*Lower 95%*	*Upper 95%*

Intercept	116.8	353.6	0.330	0.742	-592.1	825.7
Age (years)	0.7	3.0	0.239	0.812	-5.3	6.8
Fat mass (kg)	12.0	3.4	3.573	0.001	5.3	18.8
Fat-free mass (kg)	17.8	5.0	3.597	0.001	7.9	27.7
Sex	39.5	117.3	0.337	0.738	-195.6	274.6
FVC, %predicted	2.2	2.4	0.885	0.380	-2.7	7.0

Model 7 – addition of FEV_1 (%predicted) to Model 1

Regression statistics	
Multiple R	0.763
R square	0.583
Adjusted R square	0.544
Standard Error	227.933
Observations	60

ANOVA

	df	*SS*	*MS*	*F*	*Significance F*
Regression	5	3919506	783901	15.089	2.92E-09
Residual	54	2805488	51953		
Total	59	6724994			

	Coefficient	*Standard Error*	*t Stat*	*P value*	*Lower 95%*	*Upper 95%*
Intercept	323.2	308.9	1.046	0.300	-296.2	942.6
Age (years)	0.0	3.0	-0.010	0.992	-6.0	5.9
Fat mass (kg)	11.5	3.4	3.408	0.001	4.7	18.3
Fat free mass (kg)	18.3	5.0	3.685	0.001	8.4	28.3
Sex	30.5	118.9	0.257	0.798	-207.9	268.9
FEV_1, %predicted	0.3	1.9	0.138	0.891	-3.6	4.1

Model 8 – addition of FEV_1 to FVC ratio to Model 1

Regression statistics	
Multiple R	0.767
R square	0.589
Adjusted R square	0.550
Standard Error	226.378
Observations	60

ANOVA

	df	*SS*	*MS*	*F*	*Significance F*
Regression	5	3957664	791533	15.445	2.05E-09
Residual	54	2767330	51247		
Total	59	6724994			

	Coefficient	*Standard Error*	*t Stat*	*P value*	*Lower 95%*	*Upper 95%*
Intercept	625.1	392.5	1.593	0.117	-161.8	1412.0
Age (years)	-1.0	3.0	-0.319	0.751	-7.0	5.1
Fat mass (kg)	11.3	3.3	3.416	0.001	4.7	17.9
Fat free mass (kg)	18.4	4.9	3.748	0.000	8.6	28.3
Sex	11.2	118.1	0.095	0.925	-225.6	248.0
FEV_1/FVC ratio	-303.6	347.3	-0.874	0.386	-999.9	392.8

Detail statistics of cohort specific REE prediction equations by multiple linear regression modeling (least squares model). Model 1 was calculated using previously known criteria, such as age (years), sex (male=1, female=0), fat mass (kg) and fat free mass (kg). Model 2, 3, 4, 5, 6, 7, and 8 were calculated by adding asthma status (asthma diagnosis=1, no asthma diagnosis=0), FENO (ppm), white blood cell count (millions of cells), neutrophil count (millions of cells), forced vital capacity (FVC, %predicted), forced expiratory volume in 1 second (FEV_1, %predicted) or ratio FEV1/FVC to Model 1. Observations, n=60. One participant data were excluded due to not performing DEXA.

TABLE S6. Resting Energy Expenditure among Asthmatics at Pre- vs. Post-Puberty Onset of Disease

Indirect Calorimetry	Asthma Onset		*P* Value
	Pre-Puberty (n=20)	Post-Puberty (n=21)	
REE (kcals/day)	1576 ± 286	1610 ± 386	0.75
RQ	0.74 ± 0.06	0.73 ± 0.06	0.52
Prediction Equations			
MSJ (kcals/day)	1558 ± 260	1545 ± 244	0.87
%predicted	102 ± 11	104 ± 17	0.30
Residuals	-17 ± 192	-65 ± 275	0.32
Residuals (%)	-0.2 ± 13.3	-1.1 ± 19.3	0.29
HB (kcals/day)	1651 ± 282	1629 ± 266	0.85
%predicted	96 ± 10	99 ± 16	0.15
Residuals	75 ± 193	18 ± 259	0.12
Residuals (%)	5.7 ± 13.8	4.1 ± 19.7	0.15
WHO (kcals/day)	1650 ± 305	1632 ± 295	0.99
%predicted	96 ± 12	99 ± 14	0.25
Residuals	74 ± 206	22 ± 239	0.19
Residuals (%)	5.6 ± 14.6	3.9 ± 18.6	0.25
OX (kcals/day)	1596 ± 303	1576 ± 298	1.00
%predicted	100 ± 12	102 ± 15	0.19
Residuals	20 ± 202	-34.5 ± 237	0.16
Residuals (%)	2.0 ± 14.1	0.2 ± 18.0	0.19

Data represent mean ± SD.

REE, resting energy expenditure; IC, indirect calorimetry; RQ, respiratory quotient, VO2/VCO2; FFM, fat-free mass; MSJ, Mifflin-St. Jeor; HB, Harris-Benedict; WHO, World Health Organization equations; OX, Oxford equations; Residuals, difference between predicted-REE and measured-REE (residuals = $REE_{MSJ/HB/WHO/OX}$ - REE_{IC}), kcals/day. Statistical significance determined by unpaired Student's t-test or Mann-Whitney-U test when data were not normally distributed.

TABLE S7. Resting Energy Expenditure among Asthmatics with High vs. Low ACT Score

Indirect Calorimetry	High ACT score (>19, n=29)	Low ACT score (≤19, n=12)	*P* Value
REE (kcals/day)	1514 ± 236	1787 ± 462	0.02
RQ	0.73 ± 0.06	0.76 ± 0.07	0.10
Prediction Equations			
MSJ (kcals/day)	1513 ± 194	1645 ± 339	0.34
%predicted	101 ± 16	108 ± 8	0.17
Residuals	-0.2 ± 250	-142 ± 170	0.17
Residuals (%)	1.9 ± 18.6	-6.8 ± 7.2	0.16
HB (kcals/day)	1591 ± 207	1756 ± 368	0.34
%predicted	96 ± 15	101 ± 8	0.26
Residuals	77 ± 248	-31 ± 151	0.29
Residuals (%)	7.1 ± 19.2	-0.5 ± 7.9	0.38
WHO (kcals/day)	1575 ± 214	1801 ± 404	0.23
%predicted	97 ± 15	99 ± 8	0.99
Residuals	61 ± 247	15 ± 151	0.89
Residuals (%)	5.9 ± 18.9	1.8 ± 8.7	0.99
OX (kcals/day)	1519 ± 214	1746 ± 405	0.22
%predicted	101 ± 16	102 ± 8	0.83
Residuals	6 ± 246	-41 ± 142	1.00
Residuals (%)	2.2 ± 18.5	-1.5 ± 7.7	0.84

Data represent mean ± SD.

REE, resting energy expenditure; IC, indirect calorimetry; RQ, respiratory quotient, VO_2/VCO_2; FFM, fat-free mass; MSJ, Mifflin-St. Jeor; HB, Harris-Benedict; WHO, World Health Organization equations; OX, Oxford equations; Residuals, difference between predicted-REE and measured-REE (residuals = $REE_{MSJ/HB/WHO/OX}$ - REE_{IC}), kcals/day. Statistical significance determined by unpaired Student's t-test or Mann-Whitney-U test when data were not normally distributed.

TABLE S8. Resting Energy Expenditure among Asthmatics patients with and without inhaled steroid medications

**Indirect Calorimetry**	**Without Inhaled Steroids (n=17)**	**With Inhaled Steroids (n=24)**	***P* Value**
REE (kcals/day)	1533 ± 270	1637 ± 377	0.34
RQ	0.73 ± 0.05	0.74 ± 0.07	0.37
Prediction Equations			
MSJ (kcals/day)	1527 ± 225	1569 ± 267	0.60
%predicted	101 ± 16	104 ± 14	0.56
Residuals	-5.7 ± 235	-68 ± 239	0.50
Residuals (%)	1.4 ± 17.6	-2.1 ± 15.8	0.56
HB (kcals/day)	1618 ± 241	1654 ± 294	0.66
%predicted	95 ± 14	99 ± 13	0.48
Residuals	85 ± 232	18 ± 226	0.31
Residuals (%)	7.4 ± 18.1	3.1 ± 16.2	0.48
WHO (kcals/day)	1613 ± 271	1661 ± 317	0.59
%predicted	96 ± 14	99 ± 13	0.47
Residuals	80 ± 221	24 ± 225	0.30
Residuals (%)	6.7 ± 17.0	3.3 ± 16.5	0.47
OX (kcals/day)	1555 ± 270	1607 ± 318	0.59
%predicted	99 ± 15	102 ± 13	0.48
Residuals	22 ± 221	-29 ± 221	0.38
Residuals (%)	2.8 ± 16.7	-0.1 ± 15.9	0.48

Data represent mean ± SD.

REE, resting energy expenditure; IC, indirect calorimetry; RQ, respiratory quotient, VO_2/VCO_2; FFM, fat-free mass; MSJ, Mifflin-St. Jeor; HB, Harris-Benedict; WHO, World Health Organization equations; OX, Oxford equations; Residuals, difference between predicted-REE and measured-REE (residuals = $REE_{MSJ/HB/WHO/OX}$ - REE_{IC}), kcals/day. Statistical significance determined by unpaired Student's t-test or Mann-Whitney-U test when data were not normally distributed.

TABLE S9. Resting Energy Expenditure among Asthmatics patients with and without taking short acting beta agonist (SABA) medications

**Indirect Calorimetry**	**Without SABA (n=12)**	**With SABA (n=29)**	***P* Value**
REE (kcals/day)	1554 ± 265	1610 ± 366	0.98
RQ	0.75 ± 0.06	0.73 ± 0.06	0.54
Prediction Equations			
MSJ (kcals/day)	1543 ± 209	1555 ± 267	0.89
%predicted	101 ± 16	104 ± 14	0.66
Residuals	-11 ± 248	-55 ± 235	0.94
Residuals (%)	1.4 ± 19.3	-1.5 ± 15.4	0.89
HB (kcals/day)	1621 ± 233	1647 ± 288	0.83
%predicted	97 ± 15	98 ± 13	0.81
Residuals	66 ± 255	37 ± 220	1.00
Residuals (%)	6.4 ± 20.5	4.2 ± 15.6	0.86
WHO (kcals/day)	1602 ± 237	1657 ± 320	0.88
%predicted	98 ± 15	97 ± 13	0.95
Residuals	47 ± 244	48 ± 217	1.00
Residuals (%)	5.0±19.4	4.6 ± 15.7	0.75
OX (kcals/day)	1537 ± 239	1605 ± 319	0.76
%predicted	102 ± 16	101 ± 13	0.79
Residuals	-17 ± 244	-4.4 ± 213	0.87
Residuals (%)	0.8 ± 18.9	1.2 ± 15.1	0.47

Data represent mean ± SD.

REE, resting energy expenditure; IC, indirect calorimetry; RQ, respiratory quotient, VO_2/VCO_2; FFM, fat-free mass; MSJ, Mifflin-St. Jeor; HB, Harris-Benedict; WHO, World Health Organization equations; OX, Oxford equations; Residuals, difference between predicted-REE and measured-REE (residuals = $REE_{MSJ/HB/WHO/OX}$ - REE_{IC}), kcals/day. Statistical significance determined by unpaired Student's t-test or Mann-Whitney-U test when data were not normally distributed.
